# Concurrent serum lead levels and cognitive function in older adults

**DOI:** 10.3389/fnins.2023.1180782

**Published:** 2023-04-17

**Authors:** Yu Deng, Xuechun Lin, Jie Zhou, Mengchi Li, Zhenmei Fu, Dan Song

**Affiliations:** ^1^Department of Pediatric Surgery, Shandong Provincial Hospital Affiliated to Shandong First Medical University, Jinan, Shandong, China; ^2^Hubei Key Laboratory of Food Nutrition and Safety, Ministry of Education Key Laboratory of Environment and Health, Department of Nutrition and Food Hygiene, Tongji Medical College, School of Public Health, Huazhong University of Science and Technology, Wuhan, China; ^3^Department of Nursing, Shenzhen Qianhai Shekou Free Trade Zone Hospital, Shenzhen, Guangdong Province, China; ^4^School of Nursing, John Hopkins University, Baltimore, MD, United States; ^5^Department of Radiology, Shandong Provincial Hospital Affiliated to Shandong First Medical University, Jinan, Shandong, China

**Keywords:** biomarker, lead, cognition, seniors, heavy metal

## Abstract

**Introduction:**

In this study, we investigated the relationship between serum lead levels and cognitive functioning in a sample of older adults in the US.

**Method:**

Using the National Health and Nutrition Examination Survey (NHANES) 2011–2013, a total of 768 older adults aged ≥60 years were included in the analysis. Lead concentrations in the whole blood samples were assessed using mass spectrometry. We used the immediate and delayed memory portions of the Consortium to Establish a Registry for Alzheimer's Disease Word Learning Subtest (CERAD-WL), the Animal Fluency Test (AFT), and the Digit Symbol Substitution Test (DSST) to assess the participants' cognitive performance. Using sample averages and standard deviations (SDs), we computed test-specific and global cognition z-scores. To assess the relationships between the quartiles of serum lead levels and cognitive performance, we built multiple linear regression models and adjusted for covariates, including age, sex, race/ethnicity, education, depressive symptoms, alcohol usage, and body mass index.

**Results:**

The average age of the participants was 69.6 (SD 6.6) years. Approximately half of the participants were women (52.6%), non-Hispanic white (52.0%), and had completed at least some college education (51.8%). The average serum lead concentration was 1.8 g/dL (SD 1.6) for these participants. The results of multiple linear regression using individuals in the lowest serum lead quantile as a reference group revealed that the serum lead level was not associated with test-specific (CERAD-WL, AFT, and DSST) or global cognitive z-scores.

**Conclusions:**

In older adults, concurrent serum lead concentration is not related to cognitive performance. Early or continuous lead exposure may exert a greater effect on the etiology of accelerated cognitive decline with old age.

## Introduction

Lead is a naturally occurring toxic metal in the Earth's crust. Because of the massive environmental contamination caused by the widespread use of lead, lead exposure has become a serious public health problem. Humans can be exposed to lead by breathing in lead particles and consuming dust, water, and food that have been contaminated with lead. According to the World Health Organization's 2021 update on the public health impact of chemicals: knowns and unknowns, lead exposure is estimated to be responsible for a loss of 21.7 million disability-adjusted life years globally, highlighting its long-term impact on people's health. Lead has a half-life that ranges from a month in the blood to 25-30 years in the bones. Because lead builds up in the bones during a person's lifetime and because bones hold the majority of the body's burden of lead, lead in the bones is often used as a biomarker of cumulative exposure. The nervous system is particularly sensitive to lead exposure (Wani et al., 2015). Some studies have suggested that cumulative exposure to lead, as measured by the lead level in the bones, is associated with accelerated cognitive decline at an older age. However, it is presently unclear whether concurrent plasma lead levels are associated with worse cognitive function in older adults. Some studies have reported significant associations with cognitive function measured by the Mini-Mental State Examination (MMSE) (Wright et al., 2003), verbal ability, and memory (Payton et al., 1998; Weisskopf et al., 2007). However, the findings of other studies did not support these conclusions (Weisskopf et al., 2004; Shih et al., 2006; Weisskopf et al., 2007; Weuve et al., 2009).

There is a research gap on whether accumulative lead exposure, concurrent lead exposure, or both is associated with cognitive function in older adults. Thus, this study investigated the relationship between concurrent plasma lead levels and cognitive function in a nationally representative group of older participants in the National Health and Nutrition Examination Survey (NHANES) 2013–2014. The findings of this study will help elucidate the effect of concurrent exposure to lead on cognitive function among older adults.

## Methods

### The parent study design

The NHANES is a program of studies based on continuous cross-sectional surveys designed to assess the health and nutrition status of the population in America, led biannually by the National Center for Health Statistics of the Centers for Disease Control and Prevention. The NHANES program began in the early 1960s and currently focuses on various health and nutrition measurements. Instead of utilizing simple random sampling, the NHANES data were collected using a complex and multistage probability design to represent the census makeup of civilian, noninstitutionalized populations (Johnson et al., 2013). Medical, dental, and physiological measurements and laboratory tests carried out by qualified doctors, medical and health technicians, and dietary and health interviewers were used to evaluate participants' demographic, socioeconomic, dietary, and health-related statuses. In-person interviews and physical examinations were conducted at participants' residences and mobile exam centers with special settings.

The inclusion criteria in this study specified individuals who were 60 years of age or older and had available information on both their plasma lead levels and cognitive function. The NHANES 2013–2014 survey included a total of 9,813 participants. Those older than 60 (*n* = 768) and those with no available information about their plasma lead levels (*n* = 0) or cognitive performance (*n* = 0) were excluded. Eventually, this study comprised a total of 768 older adults aged 60 years and above.

### Ethical considerations

The National Center for Health Statistics Research Ethics Review Board approved NHANES.

### Measures

#### Independent variable: quartile of plasma lead level (1st quartile [reference group], 2nd quartile, 3rd quartile, and 4th quartile)

Before being sent to Georgia Regents University for testing, the samples were stored at freezing temperatures (−70°C). After a quick dilution sample preparation process, mass spectrometry was used to directly evaluate the lead content in whole plasma specimens. The diluted plasma sample in liquid form was blasted through a nebulizer, which dispersed the large droplets of liquid into an argon aerosol. A stream of flowing argon was used to selectively transport the smaller aerosol particles past the spray chamber and into inductively coupled plasma. Plasma with a temperature of 6,000–8,000 K and a predominance of positive argon ions and electrons was produced by connecting radio-frequency power into flowing argon. The complete, detailed method has been published in a previous publication (National Health Nutrition Examination Survey, 2016). This method had a limit of detection (LOD) of 0.07 μg/dL for plasma lead levels. For values below the LOD, an imputed value was filled as the analyte. This value was the LOD divided by the square root of 2. The NHANES employed several techniques to check the accuracy of the analyses carried out by the contract laboratories, including performing blind split samples obtained during “dry run” sessions in the MEC.

Moreover, 2.0% of all specimens underwent random repeat testing at contract laboratories. A review of the results was done. Uncertain values or incomplete data were forwarded to the performing laboratory for verification. In the present study, we categorized participants' plasma levels of lead into four groups based on quartile and used the 1st quartile (the lowest quartile) as a reference. This is consistent with prior NHANES-based epidemiological studies (Fu et al., 2022a,b; Li et al., 2023).

#### Dependent variable: cognitive function

The NHANES team assessed cognitive function with three cognitive performance tests, including the Consortium to Establish a Registry for Alzheimer's Disease Word Learning Subtest (CERAD-WL), the Animal Fluency Test (AFT), and the Digit Symbol Substitution Test (DSST). All the surveys were completed on the same day and were administered by two trained interviewers in a mobile examination center. The participants were given the option to take the surveys in their preferred language.

The CERAD-WL has been widely used for various clinical purposes, including assessment for Alzheimer's disease (Reid et al., 2002), identification of mild cognitive impairment, differential diagnosis of recently identified dementias, cognitive assessment of modifiable risk factors for AD, and has been used in major epidemiological surveys worldwide (Morris et al., 1989; Prince et al., 2003; Gao et al., 2009). In NHANES, CERAD-WL was used to assess the participants' immediate and delayed learning ability for new verbal information (memory sub-domain) and included an immediate recall test after each of the three successive immediate learning trials and a delayed recall test (Davis et al., 1992; Fillenbaum et al., 2008). During each of the three learning trials, 10 words appeared on the screen one at a time in large and bold letters, and the participants were asked to read the words out loud after each word appeared. Each learning trial displayed the same set of 10 words in different sequences and colors. For the immediate recall test, the participants were asked to recall as many as 10 words as possible right after each learning trial. The scores ranged from 0 to 10 for each of the three learning trials and were added up to calculate the total immediate recall score, ranging from 0 to 30 points. For the delayed recall tests, the participants were asked to recall as many words from the same 10-word list as possible after completing the other two cognitive tests (the DSST and the AFT). The number of words correctly recalled by the participant was recorded as the delayed test score, ranging from 0 to 10. The final CERAD-WL scores consist of both the immediate recall scores and the delayed recall scores.

The AFT is a test commonly used for cognition research that measures participants' categorical verbal fluency, a component of the executive function (Strauss et al., 2006). The participants were asked to name as many animals as possible in 1 min, with one point given for each unique animal identified. The participants in NHANES were given a practice verbal fluency test with the clothing category to become familiar with the rules. The AFT has been shown to differentiate between normal cognition, mild cognitive impairment, and probable Alzheimer's disease in older people with a sensitivity of 98.8% (García-Herranz et al., 2020). A score of fewer than 15 points indicates Alzheimer's disease following dementia in the memory clinic setting (Canning et al., 2004). The AFT has been used in large-scale screenings and epidemiologic studies (Clark et al., 2009).

The DSST is a paper-and-pencil cognitive test administered on a single sheet of paper that requires the participants to match symbols to numbers according to a key located on the top of the page (Jaeger, 2018). The DSST test evaluated participants' processing speed, sustained attention, and working memory (Ryan and Schnakenberg-Ott, 2003). During the DSST test, a total of 133 boxes were printed on a paper with the numbers on top of each box. The participants were asked to draw the symbols in each box corresponding to each number based on the key provided. The DSST total score was calculated using the number of correct matches, ranging between 0 and 133. The DSST has been used in large screening, epidemiological, and clinical studies (Plassman et al., 2007; Proust-Lima et al., 2007; Rosano et al., 2016).

#### Covariates

To minimize potential confounding between plasma lead levels and cognitive function, we reviewed the literature (Ge et al., 2018, 2020; Li et al., 2019; Fu et al., 2022a) and adjusted for covariates, including age (years), sex (male or female), race/ethnicity (Mexican Americans, other Hispanics, non-Hispanic white, or non-Hispanic Black), an education level (below high school, high school graduate, or some college or above), depressive symptoms, body mass index (kg/m^2^) (< 18.5, 18.5-24.9, 25-29.9, or ≥30), and alcohol use (drinks per day) (0–1, 2, or 3 and more). Depressive symptoms were measured using the Patient Health Questionnaire, whose total score ranges between 0 and 27. A higher score indicates more severe depressive symptoms (Kroenke et al., 2001).

### Statistical analysis

The averages and standard deviations (SD) of the values from the cognitive tests were used to compute the standardized z-scores for the CERAD-WL immediate recall, the CERAD-WL delayed recall, the AFT, and the DSST. The averages and SDs of the test-specific z-scores were then used for the calculation of global cognition z-scores. Multivariate linear regression models were developed to investigate the independent connection between plasma lead quartiles (reference: the 1st quartile) and test-specific and global cognitive z-scores while controlling the abovementioned confounders. We checked both the z-scores to determine if the covariates were multicollinear before building the regression models. There was no multicollinearity, as indicated by the variance inflation factor of < 10. Statistical significance was defined as a confidence interval (CI) of 95%, excluding zero. All analyses were carried out using SPSS.

## Results

Table 1 shows the characteristics of the participants. Of the 768 participants (average age 69.6; standard deviation [SD] 6.6), approximately half of the participants were women (52.6%), non-Hispanic white (52.0%), completed some college education or above (51.8%), and had a BMI of ≥30kg/m^2^ (36.8%). Their average plasma lead level was 1.8 μg/dL (SD 1.6). The participants' average CERAD-WL immediate memory, CERAD-WL delayed memory, AFT, and DSST score was 19.6 (SD 4.6), 6.2 (SD 2.4), 16.6 (SD 5.3), and 45.8 (SD 17.2), respectively.

**Table 1:** Characteristics of the participants by plasma lead level quartile.^a^

**Variables**	**Quartile 1 ≤ 0.960 ug/dl (*n =* 196)**	**Quartile 20.960–1.350 ug/dl (*n =* 189)**	**Quartile 31.350–2.065 ug/dl (*n =* 191)**	**Quartile 4>2.065 ug/dl (*n =* 192)**	**Total (*n =* 768)**
Age, years	68.3 (5.8)	69.6 (6.7)	70.7 (6.4)	69.6 (7.0)	69.6 (6.6)
**Sex, n (%)**					
Men	79 (40.3%)	85 (45.0%)	88 (46.1%)	112 (58.3%)	364 (47.4%)
Women	117 (59.7%)	104 (55%)	103 (53.9%)	80 (41.7%)	404 (52.6%)
**Race/ethnicity, n (%)**					
Mexican Americans	31 (15.8%)	22 (11.6%)	22 (11.5%)	12 (6.3%)	87 (11.3%)
Other Hispanics	22 (11.2%)	8 (4.2%)	16 (8.4%)	17 (8.9%)	63 (8.2%)
Non-Hispanic Whites	99 (50.5%)	104 (55.0%)	101 (52.9%)	95 (49.5%)	399 (52.0%)
Non-Hispanic Blacks	23 (11.7%)	33 (17.5%)	38 (19.9%)	50 (26.0%)	144 (18.8%)
Other	21 (10.7%)	22 (11.6%)	14 (7.3%)	18 (9.4%)	75 (9.8%)
**Education, n (%)**					
Below high school	58 (29.6%)	35 (18.5%)	39 (20.4%)	45 (23.4%)	177 (23.0%)
High school graduate	48 (24.5%)	52 (27.55)	37 (19.4%)	56 (29.2%)	193 (25.1%)
Some colleges or above	90 (45.9%)	102 (54.0%)	115 (60.2%)	91 (47.4%)	398 (51.8%)
**Body mass index, n (%)**					
< 18.5 kg/m^2^	3 (1.5%)	3 (1.6%)	4 (2.1%)	4 (2.1%)	14 (1.8%)
18.5–24.9 kg/m^2^	38 (19.55)	42 (22.3%)	51 (27.1%)	63 (33.3%)	194 (25.5%)
25.0–29.9 kg/m^2^	63 (32.3%)	76 (40.4%)	64 (34.0%)	69 (36.5%)	272 (35.8%)
≥30 kg/m^2^	91 (46.7%)	67 (35.6%)	69 (36.7%)	53 (28.0%)	280 (36.8%)
Plasma lead levels, μg/dL	0.74 (0.16)	1.15 (0.11)	1.66 (0.21)	3.49 (2.37)	1.76 (1.59)
Depressive symptoms	5.6 (5.3)	5.5 (5.2)	5.2 (4.9)	5.5 (4.7)	5.5 (5.0)
Alcohol use, number of drinks/day	1.7 (1.0)	1.7 (1.7)	10.8 (94.2)	2.1 (1.8)	4.1 (47.4)
CERAD W-L immediate recall	19.8 (4.7)	19.8 (4.5)	19.8 (4.6)	19.1 (4.8)	19.6 (4.6)
CERAD W-L delayed recall	6.5 (2.3)	6.6 (2.3)	6.7 (2.2)	6.2 (2.3)	6.2 (2.4)
Animal fluency test	16.9 (5.1)	18.1 (5.8)	17.7 (5.8)	16.3 (4.8)	16.6 (5.3)
Digit symbol substitution test	51.0 (18.5)	52.1 (16.2)	49.8 (17.7)	44.4 (16.1)	45.8 (17.2)

^a^Data were presented as the average (standard deviation) for continuous variables and n (%) for categorical variables.

Table 2 shows the averages and 95% CIs of the cognitive test-specific z-scores stratified by plasma level quartiles. For participants in the 1st quartile, their CERAD W-L immediate recall, CERAD W-L delayed recall, the AFT, and the DSST average z-scores were 0.03 (95% CI −0.11, 0.17), 0.03 (95% CI −0.11, 0.17), −0.01 (95% CI −0.15, 0.12), and 0.04 (95% CI −0.10, 0.19), respectively. For the participants in the 2nd quartile, their CERAD W-L immediate recall, CERAD W-L delayed recall, the AFT, and the DSST average z score were 0.03 (95% CI −0.11, 0.17), −0.01 (95% CI −0.15, 0.13), 0.05 (95% CI −0.11, 0.20), and 0.10 (95% CI −0.04, 0.24), respectively. For participants in the 3rd quartile, their CERAD W-L immediate recall, CERAD W-L delayed recall, the AFT, and the DSST average z-scores were 0.04 (95% CI −0.10, 2.020.18), 0.10 (95% CI −0.04, 0.23), 0.06 (95% CI −0.08, 0.21), and 0.05 (95% CI −0.09, 0.20), respectively. The CERAD W-L immediate recall, CERAD W-L delayed recall, the AFT, and the DSST average z-scores were −0.10 (95% CI −0.25, 0.05), −0.10 (95% CI −0.25, 0.05), −0.08 (95% CI −0.22, 0.06), and −0.18 (95% CI −0.32, −0.05), respectively, among participants in the 4th quartile. The global cognition average z–score from the lowest to the highest quartiles was 0.09 (95% CI −0.36, 0.53), 0.17 (95% CI −0.30, 0.63), 0.25 (95% CI −0.19, 0.70), and −0.46 (95% CI −0.90, −0.01), respectively.

**Table 2:** Cognitive z-scores and 95% confidence intervals by the plasma lead level quartile.

	**Quartile 1 ≤ 0.960 ug/dl (*n =* 196)**	**Quartile 2 0.960–1.350 ug/dl (*n =* 189)**	**Quartile 3 1.350–2.065 ug/dl (*n =* 191)**	**Quartile 4 >2.065 ug/dl (*n =* 192)**
CERAD W-L immediate recall	0.03 (−0.11, 0.17)	0.03 (−0.11, 0.17)	0.04 (−0.10, 0.18)	−0.10 (−0.25, 0.05)
CERAD W-L delayed recall	0.03 (−0.11, 0.17)	−0.01 (−0.15, 0.13)	0.10 (−0.04, 0.23)	−0.10 (−0.25, 0.05)
Animal fluency test	−0.01 (−0.15, 0.12)	0.05 (−0.11, 0.20)	0.06 (−0.08, 0.21)	−0.08 (−0.22, 0.06)
Digit symbol substitution test	0.04 (−0.10, 0.19)	0.10 (−0.04, 0.24)	0.05 (−0.09, 0.20)	−0.18 (−0.32, −0.05)
Global cognition	0.09 (−0.36, 0.53)	0.17 (−0.30, 0.63)	0.25 (−0.19, 0.70)	−0.46 (−0.90, −0.01)

Multiple linear regression with a reference group being those in the 1st quantile of plasma lead level showed that concurrent plasma cotinine level is not associated with test-specific (CERAD-WL, AFT, and DSST) or global cognitive z-scores (all the 95% CIs included 0) (Table 3).

**Table 3:** The independent associations of plasma lead level quartile (reference: ≤ 0.960 ug/dl) with cognitive specific test and global cognition z-scores.^a^

	**Quartile 1 ≤ 0.960 ug/dl (*n =* 196)**	**Quartile 2 0.960–1.350ug/dl (*n =* 189)**	**Quartile 3 1.350–2.065ug/dl (*n =* 191)**	**Quartile 4 >2.065 ug/dl (*n =* 192)**
CERAD W-L immediate recall	Reference	−0.037 (−0.298, 0.223)	0.054 (−0.206, 0.314)	−0.105 (0.361, 0.150)
CERAD W-L delayed recall	Reference	0.037 (−0.218, 0.292)	0.215 (−0.0390, 0.469)	−0.001 (−0.251, 0.249)
Animal fluency test	Reference	0.128 (−0.131, 0.387)	0.205 (−0.053, 0.463)	0.000 (−0.254, 0.254)
Digit symbol substitution test	Reference	−0.100 (−0.320, 0.120)	−0.003 (−0.221, 0.216)	−0.228 (−0.444, −0.013)
Global cognition	Reference	0.015 (−0.724, 0.755)	0.472 (−0.263, 1.206)	−0.334 (−1.058, 0.389)

^a^The models were adjusted for sex, race/ethnicity, education, alcohol use, and BMI.

## Discussion

This study's findings suggest that concurrent lead levels are not associated with cognitive function in older adults. However, it is possible that lead exposures during early life or over a long duration may have a greater impact on the etiology of accelerated cognitive deterioration in later years. The findings of this study contribute to the literature and add evidence to the controversial question of whether concurrent plasma lead levels are associated with cognitive function in older adults.

A growing body of evidence shows that lead is associated with worse cognitive function in older adults (Shih et al., 2006). However, it is unclear whether cumulative exposure to lead as measured by tibia lead level or recent exposure to lead as measured by plasma lead level or both are associated with cognitive function in older adults. The finding of this study is consistent with two prior studies on plasma lead levels in which the researchers did not detect a significant association between plasma lead levels and cognitive function in older adults (Shih et al., 2006; van Wijngaarden et al., 2011). In a systematic review of 21 studies contrasting and evaluating the relationships between recent (in the plasma) and cumulative (in the bone) lead exposures and neurobehavioral outcomes (Shih et al., 2007), researchers found that associations with biomarkers of cumulative dosage were higher and more consistent than those with plasma lead levels. In another meta-analysis including 22 studies, researchers found that neurobehavioral deficits existed at the current plasma lead concentration of 40 lg/100 ml (Meyer-Baron and Seeber, 2000). To summarize, findings regarding the relationship between plasma lead levels and adult neurobehavioral outcomes are controversial.

Lead is a known neurotoxin that accumulates and persists longer in the brain tissue than in other body parts. The half-life of lead in the plasma is approximately 35 days, but it lasts ~2 years in the brain tissues (Heidari et al., 2021). Lead interferes with several neurochemical pathways, such as the plasma-brain barrier capillary integrity, synaptogenesis, the formation of myelin, and catecholamine metabolism in the central nervous system (Solon et al., 2008). The biologically plausible mechanisms for the association between plasma lead and cognitive function found in other studies are complicated. First, as a redox-inactive metal, lead induces oxidative stress through depleting thiols and impairing antioxidant defense systems (Ercal et al., 2001). Excessive oxidative stress leads to endoplasmic reticulum stress, mitochondrial damage, and neuronal apoptosis (Sanders et al., 2009). Second, lead disrupts the body's homeostatic levels of essential metals and alters normal metal signaling (Zhu et al., 2013). Lead exposure results in calcium hyperactivation, which causes neurons to experience excitotoxic damage (Sanders et al., 2009). It may disrupt zinc-dependent transcription factors and modify the regulation of gene transcription (Zawia et al., 2000). Third, exposure to lead results in epigenetic modifications in the brain regions and alterations of the epigenetic regulator (Bakulski et al., 2020). Finally, lead inhibits crucial enzymes involved in the heme synthesis process (Piomelli, 2002). Anemia caused by cerebral hypoxia is also believed to greatly impair cognition (Petranovic et al., 2008). Besides, several other mechanisms have also been proposed, such as affecting neurotransmitter storage and releasing and damaging the mitochondria (van Wijngaarden et al., 2011).

The study has several advantages. First, the study population consists of a nationally representative sample of older adults and thus has good generalizability. Moreover, to minimize residual confounding, we adjusted a comprehensive list of sociodemographic, lifestyle, mental health, and physical health covariates. In addition, older adults are vulnerable to cognitive impairment; thus, this study focused on a vulnerable group of older people. The limitation of this study is mainly the cross-sectional design. As a result, we could not establish causality or assess changes in plasma lead levels and cognitive function over time.

Moreover, even though plasma lead levels have a relatively long half-life, they still only measure a person's recent exposure to lead and do not reflect his/her long-term exposure to lead. In addition, we may not have assessed all cognitive domains with three cognitive tests. Finally, the NHANES survey may not fully represent some sub-populations, such as rural populations, homeless people, and non-English speaking individuals. Future studies are expected to use longitudinal studies to examine the temporal relationship between plasma and bone lead levels and full cognitive domains in older adults, especially those from non-western countries. Such studies would shed light on whether long-term lead accumulation in the body is associated with worse cognitive function among older adults.

To conclude, in this study, we did not find an association between concurrent plasma lead levels and cognitive function in a nationally representative sample of older adults. Our finding indicates that lead exposures in early life or over a long duration may have more of an impact on the etiology of accelerated cognitive deterioration in older adults.

## Data availability statement

The original contributions presented in the study are included in the article/supplementary material, further inquiries can be directed to the corresponding author.

## Ethics statement

The studies involving human participants were reviewed and approved by the National Center for Health Statistics Research Ethics Review Board approved the NHANES. The patients/participants provided their written informed consent to participate in this study. Written informed consent was obtained from the individual(s) for the publication of any potentially identifiable images or data included in this article.

## Author contributions

YD, ML, XL, ZF, and DS designed this study, drafted the initial manuscript, and searched for literature. JZ conducted the statistical analysis. All authors critically revised the manuscript and approved the final version of the manuscript.
